# Distinct Cleavage Trajectories Mediated by DNA and RNA Substrate in Argonaute From *Methanocaldococcus Fervens*

**DOI:** 10.1002/advs.77620

**Published:** 2026-09-08

**Authors:** Fuyong Huang, Taiyu Chen, Meng Fang, Qinmiao Duanmu, Kangle Mu, Yuqi Bi, Siwei Xu, Xin Tao, Jiening Wang, Shan Wu

**Affiliations:** ^1^ Hubei Collaborative Innovation Center for Green Transformation of Bio‐Resources Hubei Key Laboratory of Industrial Biotechnology School of Life Sciences Hubei University Wuhan Hubei China

**Keywords:** argonaute, biochemistry, biology, dna, nuclease, nucleic acid, rna, tetrad, thermophile

## Abstract

Prokaryotic Argonautes (pAgos) have emerged as highly versatile, programmable nucleases, while the full exploitation of their biotechnological utility still requires a deeper understanding of their catalytic mechanisms. Here, we characterize a thermophilic Argonaute from *Methanocaldococcus fervens* (MfAgo), which demonstrates diverse substrate targeting and high catalytic efficiency across a broad temperature range. Notably, MfAgo exhibits exceptional accuracy during RNA cleavage, and structural analysis of MfAgo in various states throughout the process reveals a stepwise conformational rearrangement, highlighting the necessity of dimerization for the stabilization of the catalytic tetrad. In contrast, cleavage assays reveal that DNA cleavage by MfAgo is robust and independent of dimerization. Furthermore, we identified an additional nucleic acid binding pocket on the surface of MID domain, which may mediate the temperature‐dependent activity. Collectively, these findings establish MfAgo as a highly versatile and promising tool for biotechnology applications.

## Graphical abstract

This study characterizes MfAgo, an enzyme exhibiting highly efficient DNA‐guided DNA and RNA cleavage capability. Precise RNA cleavage strictly requires dimerization to stabilize the PIWI‐loop region, while its robust DNA cleavage functions in a dimerization‐independent manner. Furthermore, an additional nucleic acid‐binding pocket forms a “mismatch‐interlock” conformation, which may be associated with its temperature‐dependent cleavage activity.

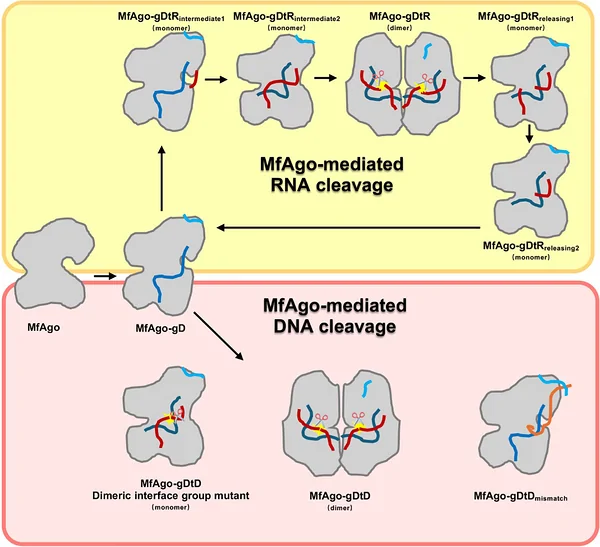

## 1 Introduction

Argonaute proteins (Agos) are distributed across all three domains of life [1]. Eukaryotic Argonautes (eAgos) are the core catalytic components of the RNA‐induced silencing complex (RISC), mediating RNA interference (RNAi) [2]. In contrast, prokaryotic Argonautes (pAgos) exhibit broader functional diversity, while they primarily utilize small DNA guides to target DNA or RNA substrates [3]. This substrate diversity highlights their potential as versatile tools for genome editing and nucleic acid detection.

According to structural diversity, pAgos can be categorized into three main groups including long‐A, long‐B and short Agos [4]. Unlike long‐A Agos, long‐B and short Agos lack the capability of direct cleavage due to substitution within the essential catalytic tetrad. Long‐A Agos adopt a conserved bi‐lobe conformation similar to eAgos, comprising an N‐terminal lobe (N‐terminal and PAZ domain), a C‐terminal lobe (MID and PIWI domain) and connecting linkers (L1 and L2) [5]. During the cleavage, the MID and PAZ domains anchor the 5’ and 3’ ends of the guide strand, respectively. Substrate cleavage is executed by the RNase H‐like fold within the PIWI domain, after the formation of the stable guide‐target duplex. Subsequently, the N‐terminal domain facilitates duplex unwinding to promote the release of the cleavage products [6]. Recently, structural studies have revealed that, in certain pAgos, homodimerization in binary state or ternary states may represent a functionally relevant conformational transition for cleavage‐capable state or intermediate adjustment [7, 8, 9, 10].

The pAgo system has emerged as a cutting‐edge molecular tool, offering high sensitivity and sequence‐specific recognition for feasible diagnostic platforms [11, 12, 13]. To date, pAgo‐based applications have primarily relied on a few well‐characterized members, notably the thermophilic Argonautes from *Thermus thermophilus* (TtAgo) and *Pyrococcus furiosus* (PfAgo) [14, 15, 16, 17, 18]. These proteins are mainly capable of DNA‐guided cleavage of single‐stranded DNA and plasmid DNA [19, 20, 21, 22]. However, the broader utility of the pAgo system remains hindered by its limited RNA cleavage capabilities and the requirement for high reaction temperatures. While TtAgo can perform DNA‐guided RNA cleavage at elevated temperatures, it exhibits notable tolerance for mismatches, which potentially compromises the targeting precision [6, 23]. Similarly, though recent engineering of PfAgo mutants has lowered its operational temperature and enabled RNA cleavage activity, these mutants still retain significant mismatch tolerance [24].

**FIGURE 1:**
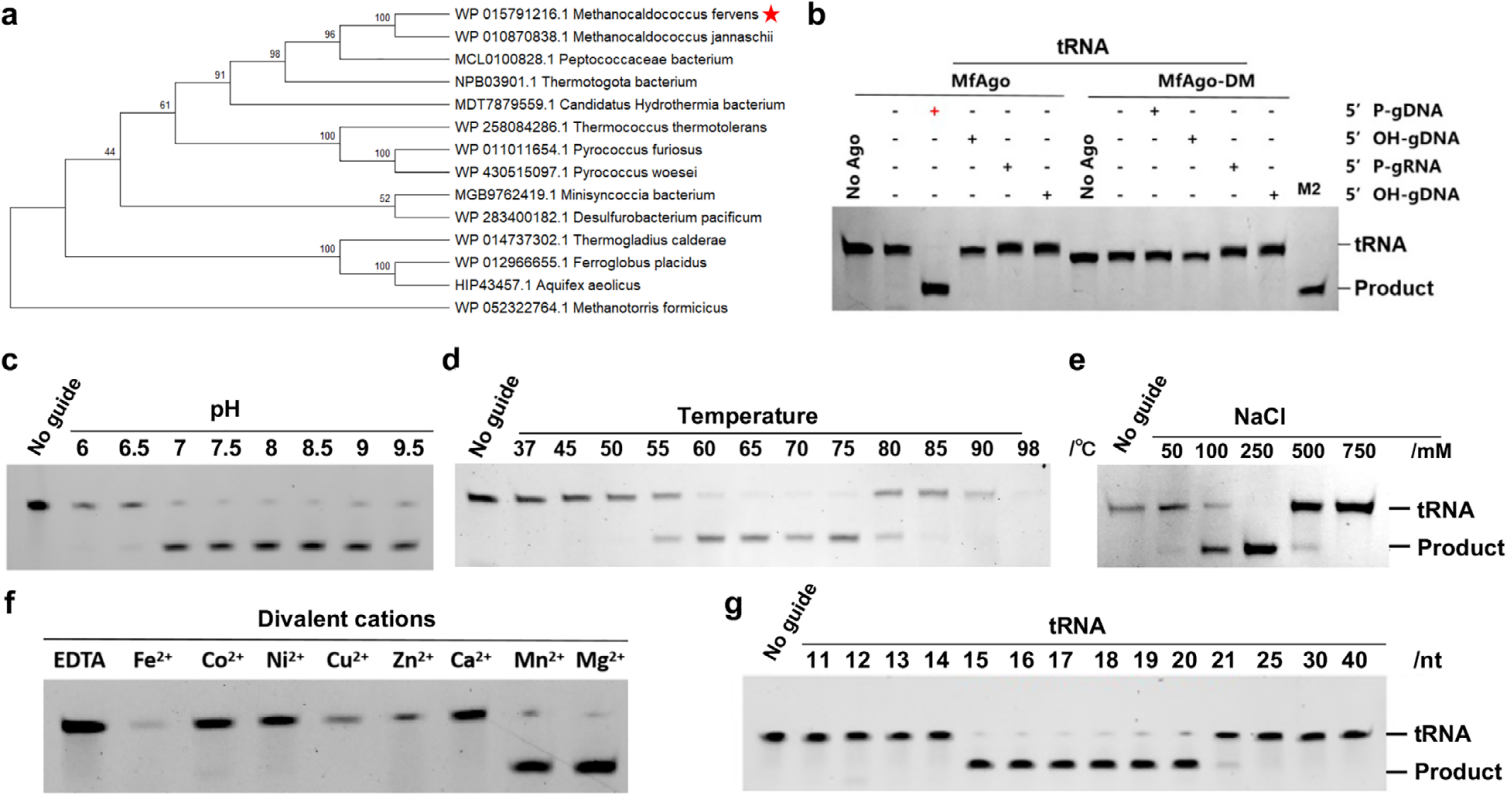
MfAgo exhibits DNA‐guided RNA cleavage activity (a) Schematic phylogenetic tree of MfAgo based on amino acid sequence alignment. (b) RNA cleavage assays of MfAgo guided by 5’‐modified DNA or RNA at a molar ratio of MfAgo:guide:target (10:2:1). A reaction lacking guide DNA served as a negative control; M2 denotes a synthetic 5’‐FAM‐labeled 34 nt RNA product. MfAgo‐DM, designated as the D497A/D563A double mutant, abrogates catalytic activity by introducing alanine substitutions at two positions within the catalytic tetrad. (c) pH dependence of MfAgo‐mediated RNA cleavage, examined over a pH range of 6.0 to 9.5. The upper bands correspond to uncleaved RNA substrates, and the lower bands to cleaved products. (d) Temperature dependence of MfAgo‐mediated RNA cleavage, assayed from 37°C to 98°C for 20 min. (e) NaCl concentration dependence of MfAgo‐mediated RNA cleavage, tested from 50 to 750 mM NaCl. (f) Divalent cation dependence of MfAgo‐mediated RNA cleavage, assayed in the presence of 5 mM of the indicated divalent cations. (g) Guide length dependence of MfAgo‐mediated RNA cleavage, performed with guide DNAs of varying lengths in optimized RNA cleavage buffer (10 mM Hepes, pH 7.5, 250 mM NaCl, 5% Glycerol, and 5 mM MgCl_2_) at 60°C for 20 min.

To further address these limitations, we conducted a phylogenetic analysis of long‐A Agos and identified a candidate from the hyperthermophilic archaeon *Methanocaldococcus fervens* (MfAgo) [25]. MfAgo shares a close evolutionary relationship with the well‐studied PfAgo and MjAgo (*Methanocaldococcus jannaschii*) (Figure 1a). Our preliminary findings demonstrate that MfAgo retains robust DNA‐guided DNA cleavage activity comparable to TtAgo and PfAgo. Notably, we identified that MfAgo possesses an additional RNA cleavage across a broad temperature range (55°C to 80°C). The combination of substrate versatility and thermal adaptability positions MfAgo as a highly promising programmable nuclease for next‐generation nucleic acid detection technologies.

To elucidate the cleavage mechanism of MfAgo and facilitate its development as a versatile programmable nuclease, we determined a series of MfAgo structures in distinct functional states at resolutions ranging from 2.8 to 3.4 Å. Structure‐based mutation analysis together with biochemical studies reveal key elements underlying the endonuclease activity of MfAgo between DNA and RNA. Notably, we observed an additional nucleic acids‐binding pocket within the MfAgo structure, proposing it as a temperature‐related element linked to cleavage capability. Collectively, our findings provide a comprehensive understanding of the cleavage capability and mechanism of MfAgo, thereby establishing a rational framework for the engineering of thermophilic Agos.

## 2 Results

### 2.1 Substrate Versatility and Cleavage Requirements of MfAgo

To systematically evaluate the substrate spectrum of MfAgo, we performed in vitro cleavage assays and discovered that MfAgo efficiently cleaves single‐stranded RNA (ssRNA) substrates when directed by 5’‐phosphorylated (5’‐P) DNA guides at elevated temperature, in addition to its canonical DNA cleavage activity (Figure 1b and Figure S1a,b). This unexpected dual‐substrate versatility highlights its potential utility in diverse nucleic acid manipulation applications.

Further investigations determined the optimal conditions for MfAgo‐mediated RNA cleavage and revealed that MfAgo exhibits robust activity in a slightly alkaline environment, with a pH range of 7.0 to 9.5 and over a broad temperature range of 55°C to 80°C. (Figure 1c,d) Similar to other well‐characterized pAgos, MfAgo strictly requires divalent cations (Mn^2+^ and Mg^2+^) to catalyze both DNA and RNA cleavage (Figure 1f). However, while ssDNA cleavage exhibits broad salt‐tolerance, the highest efficiency for ssRNA cleavage is achieved at 250 mM NaCl (Figure 1e and Figure S1c). Regarding substrate‐specific guide length preference, MfAgo accepts a wide range of guide lengths for DNA cleavage, whereas RNA cleavage is restricted to shorter DNA guides of 15–20 nucleotides (nt) (Figure 1g and Figure S1d).

To assess the cleavage accuracy of MfAgo, we introduced a series of DNA guides containing single‐nucleotide mismatches and evaluated their impact on cleavage efficiency. Our results demonstrate that mismatches located at the 5’‐end (position1) and 3’‐tail (position 16–18) regions minimally affected cleavage activity for both DNA and RNA target (Figure S1f). However, during the RNA cleavage, mismatches at any other central region resulted in a drastic reduction of cleavage. In contrast, DNA cleavage exhibited a higher overall mismatch tolerance, only with severe catalytic impairment occurring when the mismatches positioned near the canonical cleavage site (Figure S1f). These findings demonstrate that MfAgo‐mediated RNA cleavage possesses high sequence fidelity, suggesting MfAgo as a highly specific and programmable tool for RNA targeting.

### 2.2 Capability of Highly Structured Nucleic Acid Cleavage by MfAgo

**FIGURE 2:**
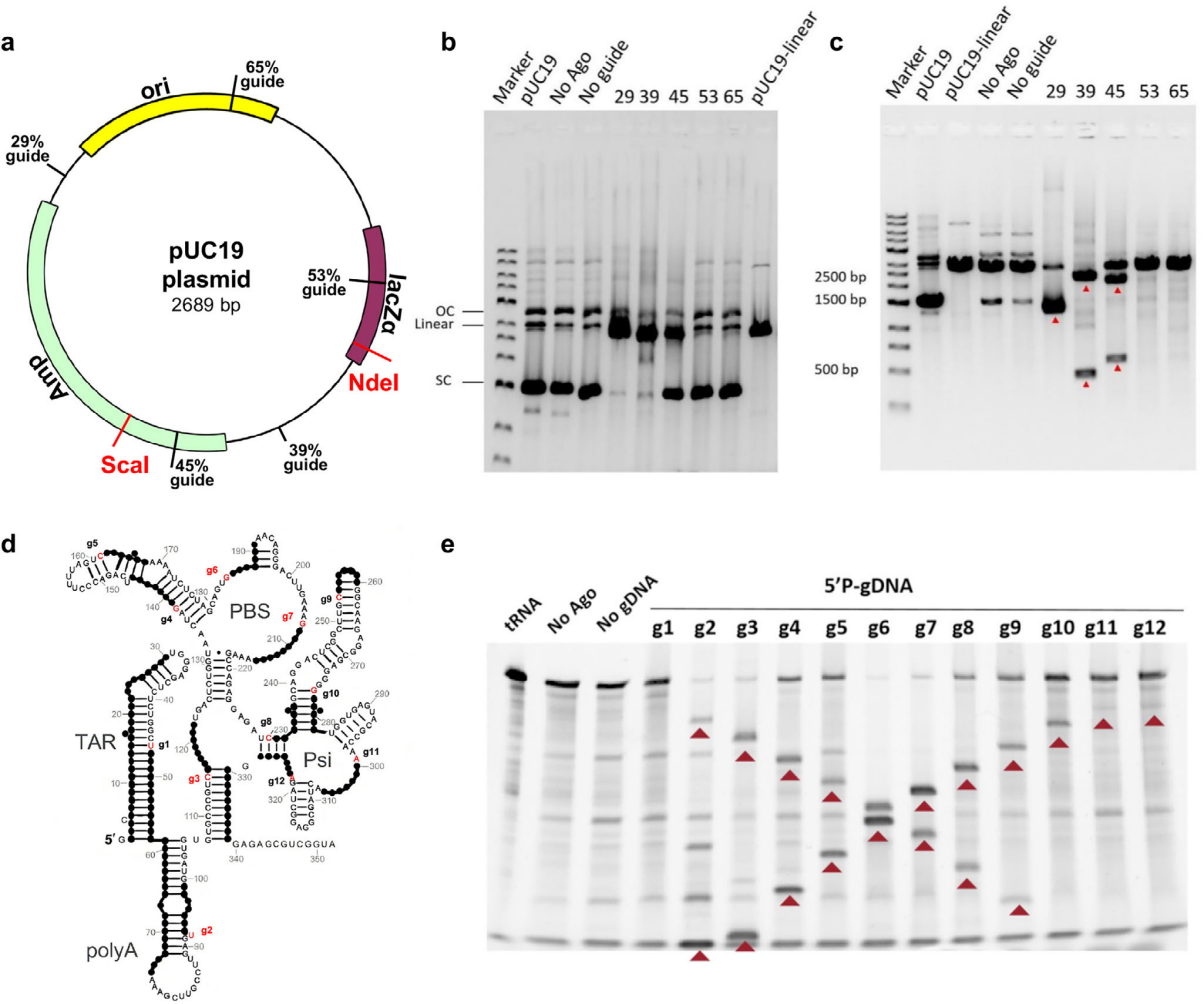
MfAgo‐mediated cleavage of the plasmid substrate and the highly structured RNA (a) Schematic of the pUC19 plasmid substrate and guide‐targeting sites. DNA guides were designed to target five regions with different GC contents (29%, 39%, 45%, 53%, and 65%). Target positions are marked in black, and the percentage above each guide pair denoted the GC content of the corresponding target region. For example, the 29% guide pair consists of forward‐ and reverse‐strand guides targeting the region with a GC content of 29%. Guide DNA sequences are listed in Table S1. ScaI and NdeI restriction sites are shown in red and were used in subsequent restriction digestion assays to verify cleavage accuracy. (b) Effects of target‐region GC content on MfAgo‐mediated plasmid cleavage. Cleavage reactions were performed with guide pairs targeting pUC19 regions of indicated GC contents. Untreated pUC19, and the reactions lacking MfAgo or guide DNA, served as controls. OC and SC represent open‐circular and supercoiled DNA, respectively. Linearized pUC19 serves as a migration reference for the full‐length linear cleavage product. (c) Validation of MfAgo‐mediated plasmid cleavage sites by restriction enzyme digestion. Cleavage products generated with guide pairs targeting the indicated GC content regions were digested with ScaI and NdeI. Red arrows indicate fragments corresponding to the predicted restriction products, supporting cleavage at the intended position. (d) Secondary structure of the HIV‐1 ΔDIS 5′UTR RNA. Twelve guide DNAs targeting distinct secondary structural sites are shown adjacent to their target sequences, with the 5’‐end of each target highlighted in red. Preferred cleavage sites (determined by assays in Figure 2e) are labelled in red. Key structural features, including TAR, ployA, PBS, and Psi, are indicated. (e) Cleavage assays of structured HIV‐1 RNA using guides targeting distinct structural motifs. tRNA, and the cleavage reactions lacking MfAgo or guide DNA are served as controls. Red arrows indicate product bands corresponding to the predicted cleavage positions.

To date, all characterized pAgos lack intrinsic helicase activity, typically relying on accessory proteins or elevated temperature to facilitate double‐stranded DNA (dsDNA) unwinding for target cleavage [26]. Consequently, the stability of the DNA duplex, which is largely dependent on the local GC content, may influence the efficiency of dsDNA cleavage. Indeed, previous studies have shown that both the guide‐independent chopping activities and the guide‐directed cleavage activity of pAgos, such as PfAgo, and TtAgo, are sensitive to the GC content of the target region [8, 27, 28]. Therefore, to evaluate whether MfAgo is suitable for precise dsDNA manipulation and its preference for target regions with different GC contents, we designed pairs of forward and reverse DNA guides targeting regions of the pUC19 plasmid with varying GC contents (Figure 2a). Utilizing the optimized conditions established for single‐stranded substrates (85°C in reaction buffer containing 10 mM Hepes, pH 7.5, 250 mM NaCl, 5% (w/v) Glycerol and 5 mM MnCl_2_), we found that MfAgo generates linear plasmid products in a strictly guide‐dependent manner. Efficient dsDNA cleavage occurred at target regions with GC contents of 29%, 39% and 45%, with peak efficiency observed at 39% GC (Figure 2b and Figure S2a). Conversely, cleavage efficiency was dramatically diminished at GC contents exceeding 45% (Figure 2b). Subsequent restriction enzyme digestion using NdeI and ScaI yielded cleaved fragments consistent with predicted sizes, confirming the accurate sequence‐specific targeting of the plasmid by MfAgo (Figure 2c). We further demonstrated the optimal parameters for plasmid cleavage and the highest degradation efficiency was achieved with guide pre‐incubation temperature between 55°C to 70°C, followed with a cleavage reaction at 60°C to 85°C (Figure S2b,c). Additionally, the generation of linear products was notably enhanced at NaCl concentration above 150 mM and required Mn^2+^ concentration around 0.1 to 1 mM (Figure S2d,e). Finally, we optimized the reaction stoichiometry through activity assays and thus established that MfAgo exhibits an enhanced cleavage efficiency at a protein:guide:target molar ratio of 3:2:1 (µM). This optimized condition provides a robust and reproducible protocol for MfAgo‐mediated dsDNA application (Figure S2f). For practical application, we developed MfAgo‐based protocols that enable precise in vitro gene manipulation. When programmed with specifically designed DNA guides, MfAgo rapidly cleaves plasmid targets into defined dsDNA fragments, which can be readily integrated into recombinant plasmids via standard DNA ligation techniques or verified by downstream fluorescent detection (Figure S3). The rapid cleavage efficiency and high accuracy of MfAgo highlights its immense potential as a robust nucleic acid manipulation tool.

Moreover, we further explore whether MfAgo possess the ability to target highly structured RNA molecules. Given the essential roles of such complex RNA structures in genome replication of diverse RNA viruses, we in vitro transcribed a highly structured 5’‐untranslated region (5’UTR) of HIV‐1 (HIV‐1 ΔDIS 5’UTR) as a model substrate [29]. This RNA substrate features a complex topology composed of diverse structural sub‐domains, including varied lengths of helices, hairpin loops and single‐stranded regions. To systematically evaluate MfAgo‐mediated cleavage across these elements, we designed a series of 16 nt DNA guides targeting each distinct secondary structure (Figure 2d, Table S1). Cleavage assays demonstrated that MfAgo loaded with DNA guides, such as g2, g3, g6 and g7, presents robust activity at 60°C for 20 min, underscoring its potential as a programmable tool for advanced RNA targeting and viral genome interference (Figure 2e).

### 2.3 Overall Architectures of MfAgo in Distinct Targeting States

**FIGURE 3:**
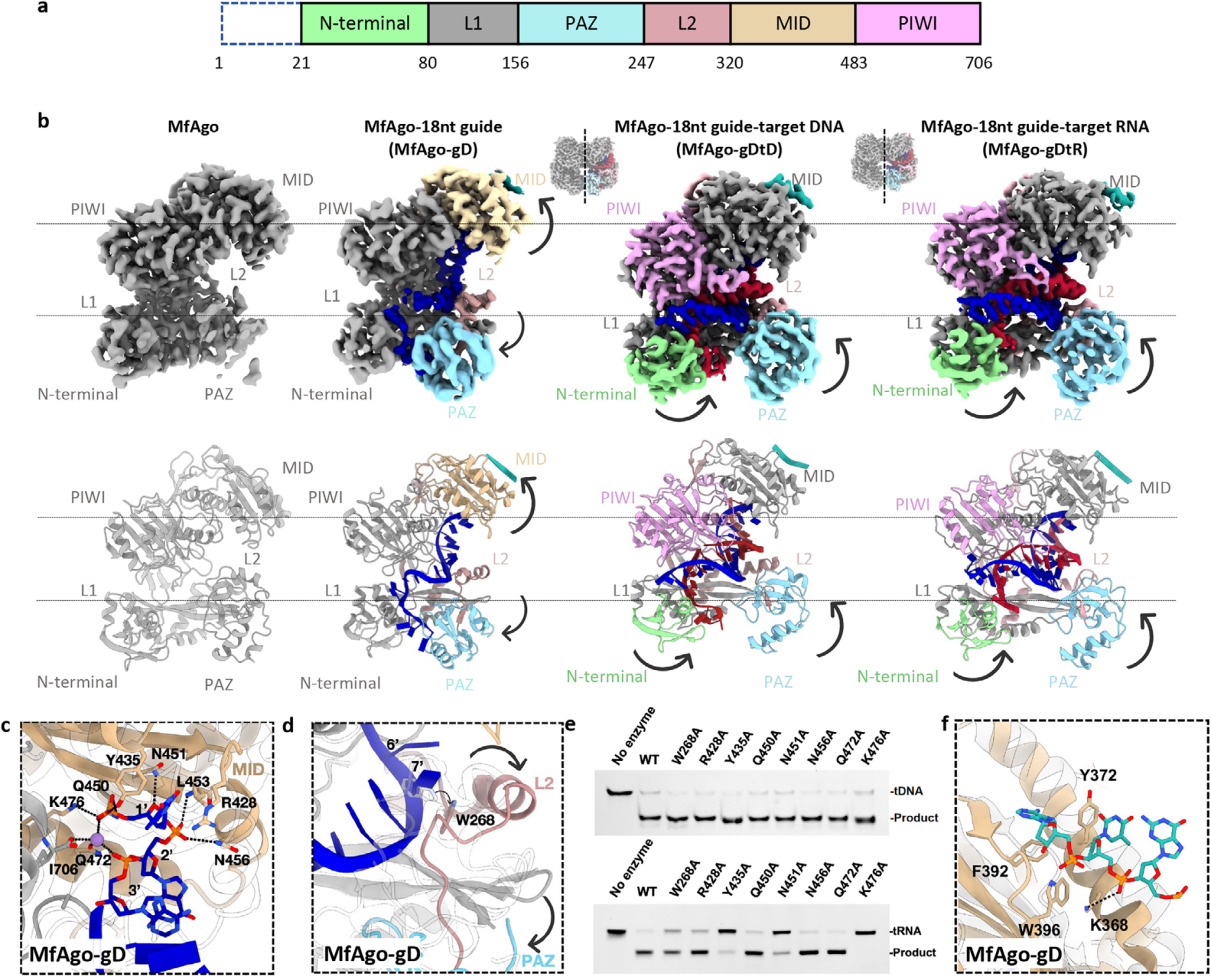
Overall architectures of MfAgo‐related complexes (a) Schematic domain organization of MfAgo. Amino acid residue ranges for each domain are indicated, and dashed lines denote regions lacking cryo‐EM density. (b) Cryo‐EM density maps and atomic structures of MfAgo in the apo, binary and ternary (with DNA or RNA target) states. Domains exhibiting conformational changes relative to the preceding state are colored according to the scheme in Figure 3a: the binary complex is compared with the apo state and each ternary complex is compared with the binary complex. Arrows indicate the direction of domain movement. The MfAgo‐gDtD and MfAgo‐gDtR ternary complexes are shown in cross‐sectional views, with the corresponding section plane illustrated in the upper right insets. (c) Detailed interactions between the MID domain and the 5’‐end of the guide DNA. The apo structure of MfAgo is superimposed and shown as a gray ribbon for comparison. (d) Close‐up view of structural rearrangements induced by guide binding. Compared with the apo state, guide binding induces an outward shift of the L2 linker and a rotation of the PAZ domain in the MfAgo‐gD complex. Arrows indicate the direction of these conformational changes. (e) Cleavage activity of wild‐type (WT) MfAgo and its mutants against DNA and RNA targets. (f) Identification of the additional nucleic acid‐binding pocket adjacent to the MID domain compared with the apo state.

To elucidate the cleavage mechanism of MfAgo and facilitate its rational engineering, we determined cryo‐EM structures across distinct functional states, including apo state (MfAgo), binary guide DNA‐loaded complex (MfAgo‐gD) and ternary target‐bound complexes with either DNA or RNA substrates (MfAgo‐gDtD or MfAgo‐gDtR), at resolutions of 3.2, 3.3, 2.8, and 2.9 Å, respectively (Figure 3b and Figures S4–S6). The high quality of the cryo‐EM density maps enabled accurate model building for all states.

Structural comparisons across these states reveal that MfAgo undergoes conserved conformational transition during guide association (Figure 3b). Upon guide association, the MID domain undergoes an outward rotation to establish a canonical interaction network with the 5’‐P guide DNA. Concurrently, the L1 linker and the PAZ domain tilt inward to anchor the 3’‐end of the guide (Figure 3c,d). Overall, MfAgo‐gD closely resembles the DNA‐guided MjAgo structure (PDB:5J5T, r.m.s.d. 1.0 Å) [30] but adopts a considerably more relaxed conformation than the binary structure of CpAgo (PDB:9JH7, r.m.s.d 1.2 Å) [8] (Figure S7a,b). Notably, the rotation of W268 in the L1 linker appears to facilitate the inward movement of the L1‐PAZ domain, providing sufficient space for the guide DNA binding. This feature distinguishes MfAgo from most other pAgos, which typically harbor residues with shorter side chains at the corresponding position, such as leucine or isoleucine (Figure 3d and Figure S7c). Cleavage assays of these key residues at the guide‐interaction interface (W268, R428, Y435, Q450, N451 and K476) revealed that while DNA cleavage exhibits much higher tolerance to these mutations, RNA cleavage is partially abolished, with the most pronounced effects observed for the Y435A, N451A and K476A mutants, underscoring the stringent structural requirements for RNA targeting (Figure 3c,e). However, mutations of residues N456 and Q472, which coordinate the second nucleotide of the guide strand, only slightly decrease and largely retained the ssRNA cleavage efficiency (Figure 3c,e). Furthermore, we observed an additional density above the MID domain in the MfAgo‐gD map, which we modeled as three extra nucleotides (Figure 3f and Figure S7d). This density, which is absent in the apo state but consistently observed in both binary and ternary complexes, is hypothesized to arise from a random fragment of the DNA guide stabilized by *π*–*π* stacking interactions (Figure 3f). An extra density has been recently reported in the binary complex of MbpAgo and similarly contributed to a guide‐derived fragment, however, this density is absent in the ternary complex of MbpAgo, highlighting a mechanistic distinction from MfAgo [10]. The presence of an extra density comparable to that of MfAgo in the ternary structure of PfAgo raises the possibility that auxiliary nucleic acid anchoring represents a conserved structural but mechanistic diverse feature among certain pAgos [7] (Figure S7d).

Both ternary complexes, MfAgo‐gDtD and MfAgo‐gDtR, adopt a dimeric conformation. Subunit alignment shows that target‐substrate pairing induces release of the 3’end of the guide strand from the PAZ domain. Simultaneously, the L2 and PAZ domains of MfAgo flip outward to accommodate the substrates, while the N‐terminal domain rotates inward to stabilize the guide‐target nucleic acid duplex (Figure 3b). Notably, this outward rotation of the PAZ domain exposes the original 3’‐end guide DNA binding pocket, which is subsequently occupied by insertion of residue F30 from the N‐terminal domain of the adjacent subunit (Figure S7e,f). During the process, the PIWI domain in both MfAgo‐gDtD and MfAgo‐gDtR undergoes a conformational rearrangement that switches the catalytic tetrad into its functional conformation, leading to cleavage between the 10 and 11 nucleotides of the target strand. This dimerization is consistent with functional transitions reported for other long pAgos, such as PfAgo (PDB:8JPX, r.m.s.d 1.1 Å) and CpAgo (PDB:9JH9, r.m.s.d 1.3 Å) (Figure S7g,h). Notably, while DNA‐targeting datasets produced only dimeric particles, classification of RNA‐targeting datasets revealed a mix of monomeric and dimeric states, indicating that the differences between MfAgo‐mediated DNA and RNA targeting require further investigation (Figure S6).

### 2.4 The Complete MfAgo‐Mediated RNA Cleavage Cycle: Dimerization of the Ternary Complex is Required

**FIGURE 4:**
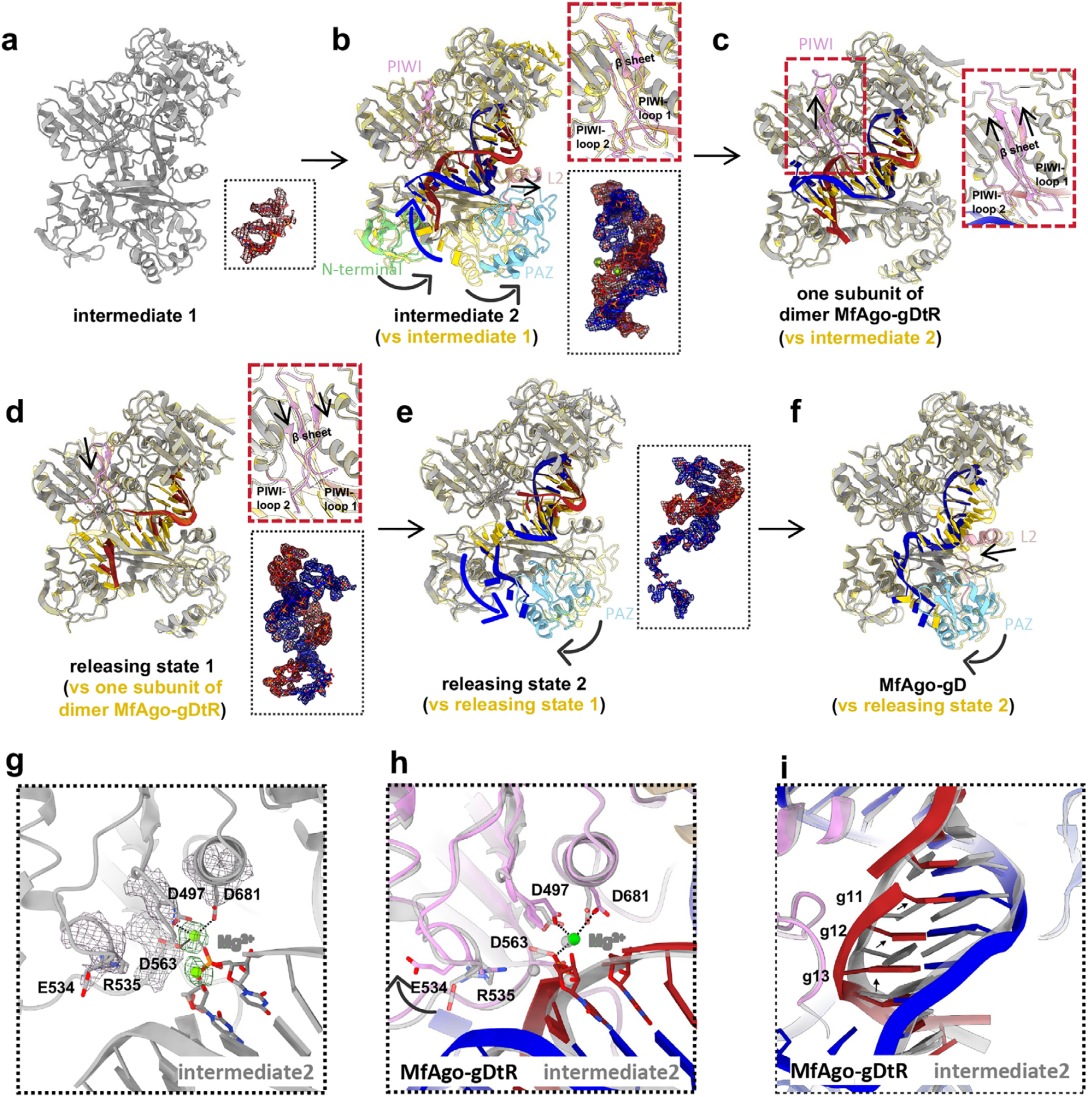
Stepwise conformational rearrangements during RNA cleavage (a–f) Cryo‐EM structures representing different states of MfAgo‐mediated ssRNA cleavage. Domains are colored according to the scheme in Figure 3a to highlight conformational transitions between successive states as indicated below. Arrows indicate the direction of domain movement. Cryo‐EM density maps for the guide and target strands are shown in black dashed boxes. A close‐up view of the PIWI loop region, comprising two loops and one β‐sheet, is shown in the red dashed box. (g) Close‐up view of the interaction network within the catalytic pocket in Intermediate 2. Cryo‐EM densities corresponding to the catalytic tetrad and cations are shown as mesh. Cations are represented as green spheres. (h) E534 exhibits an outward shift in the MfAgo‐gDtR dimeric conformation compared to the intermediate 2. (i) Compared with intermediate 2, the cleaved RNA product adopts an outward movement in the MfAgo‐gDtR complex. Arrows indicate the direction of product movement.

During cryo‐EM data processing of the ternary complex engaged in RNA cleavage, multiple monomeric states were captured in addition to the dimeric conformation. These monomeric states likely represent transient intermediate states along the RNA cleavage pathway (Figure S6). A monomeric state, designated as intermediate 1 (MfAgo‐gDtR_intermediate1_, 3.2 Å), is highly similar to MfAgo‐gD yet contains an additional three‐nucleotide pairing at guide positions g2‐g4 (Figure 4a), suggesting that it represents the initial stage of target loading. In contrast, intermediate 2 (MfAgo‐gDtR_intermediate2_, 3.4 Å), a monomeric ternary complex featuring a complete nucleic acid duplex, exhibits an outward shift of the L2 helix and the PAZ domain coupled with a slight movement of the N‐terminal domain relative to intermediate 1. These coordinated motions facilitate both the release of the guide strand's 3’ end and the propagation of the nucleic acid duplex (Figure 4b). In intermediate 2, the target RNA remains intact, and the overall structure closely resembles the single subunit of the MfAgo‐gDtR dimer, except for a subtle displacement in the PIWI‐loop region (G495‐P537, encompassing two loops and one β‐sheet) (Figure 4c). Further analysis of the intermediate 2 reveals that while the DDD residues of the catalytic tetrad and two Mg^2+^ ions are already positioned within the catalytic region, the crucial glutamate finger E534 still remains pointing outward in an uninserted conformation (Figure 4g,h), indicating a pre‐cleavage state. Furthermore, we observed an outward displacement of target nucleotide 11 during the cleavage reaction, suggesting that the dimeric MfAgo‐gDtR complex likely represents a pre‐release state occurring immediately after cleavage (Figure 4i). To address the role of the dimerization, we introduced mutations at the dimer interface between the MID‐PIWI and N‐PAZ domains of the MfAgo‐gDtD complex, generating two mutants (Group mutant 1 formed by R405A, K412A, N444A, E546A, T547A and E554A, Group mutant 2 formed by F30A, E32A, Q50A, K186A, Y188A, K191A, I238A, P239A, Y240A and P241A) (Figure S8a). Native gel analysis and size‐exclusion chromatography (SEC) analysis confirmed that the ternary complexes of these two mutants no longer adopt the dimeric form (Figure S8b,g) and AlphaFold3 predictions confirmed that the overall conformations of these two mutants structurally resemble the single subunit of the ternary complex (Figure S8d). Cleavage assays reveal the complete loss of RNA cleavage among both group mutant 1 and 2 (Figure S8c). These features imply that dimerization is strictly required to induce the final structural rearrangement to stabilize the complete catalytic tetrad, including the sequential shift of PIWI‐loop region along the β‐sheet.

Concurrently, two distinct product‐release monomer conformations were identified, designated release‐state 1 (MfAgo‐gDtR_releasing1_, 3.3 Å) and release‐state 2 (MfAgo‐gDtR_releasing2_, 3.1 Å). Release‐state 1 is structurally similar to the single subunit of MfAgo‐gDtR, except for a rearrangement of the PIWI‐loop region that resembles intermediate 2, and the loss of most density corresponding to the 5’ end of the cleaved RNA product (Figure 4d). In contrast, release‐state 2 exhibits complete loss of the 5’‐cleaved RNA product (Figure 4e). Both release states share increased flexibility in the PIWI‐loop 1 region (G495‐V508). Comparative analysis reveals that release‐state 2 additionally features a rearrangement of the PIWI region and an inward shift of the PAZ domain, which together facilitate re‐anchoring of the guide DNA 3’ end (Figure 4e). Furthermore, release‐state 2 is highly similar to the initial binary state, with only a slight tilt of the L2 helix (Figure 4f). Together, these observations establish a comprehensive pathway for the MfAgo‐mediated RNA cleavage and importantly, demonstrate that dimerization is essential for the stabilization of both PIWI‐loop1 region and the catalytic tetrad for the accuracy cleavage.

### 2.5 MfAgo Exhibits Distinct Mechanisms for DNA and RNA cleavage: Ternary Complex Dimerization is Dispensable for MfAgo‐Mediated DNA Cleavage

**FIGURE 5:**
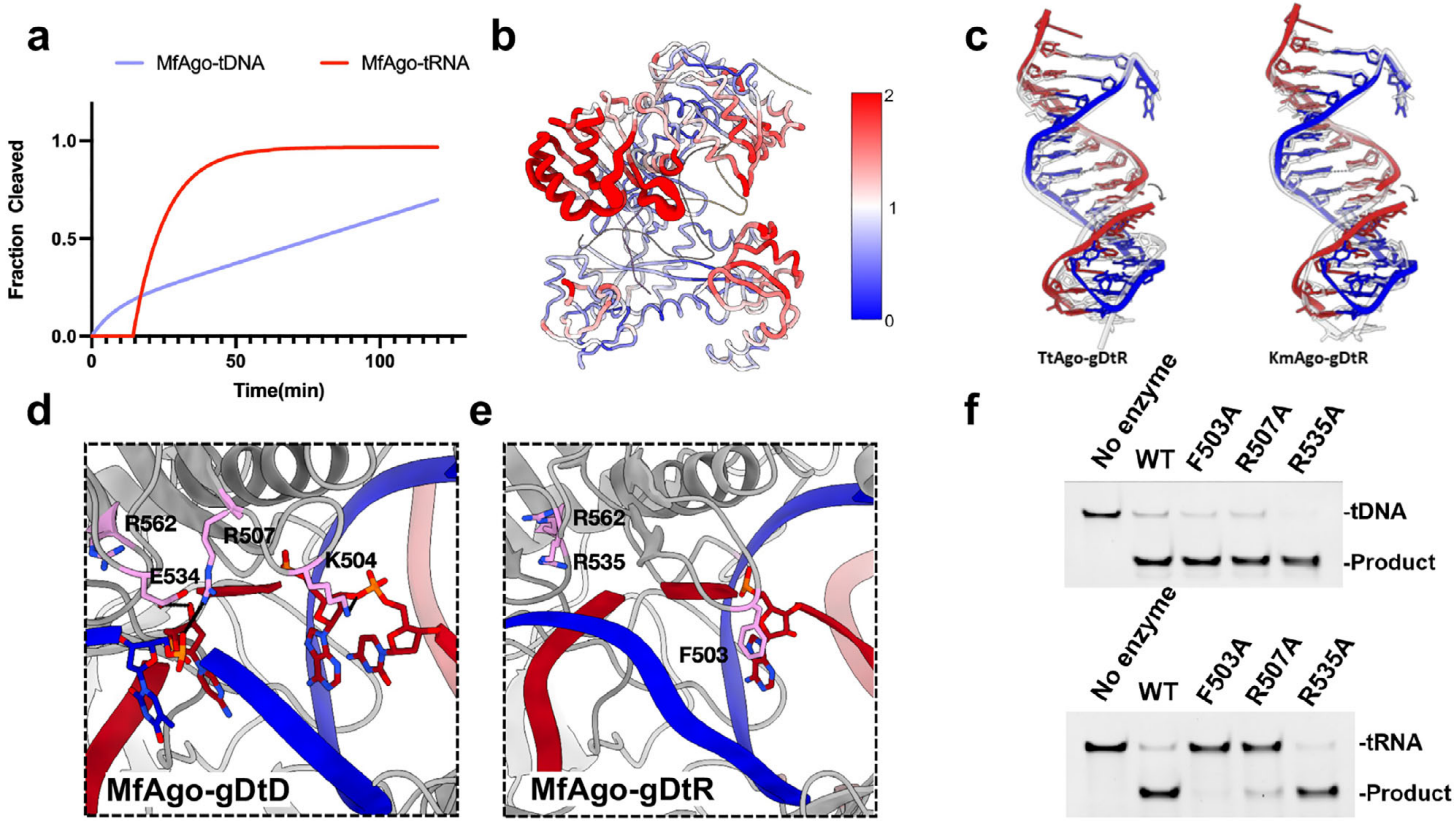
Distinctions between DNA and RNA cleavage coupled with dimerization analysis (a) Multi‐turnover kinetic analysis of DNA and RNA cleavage. Reactions were performed at a molar ratio of MfAgo:gDNA:target (DNA or RNA) of 1:2:5 (300 nM: 600 nM: 1500 nM) (b) Structural alignment of the MfAgo‐gDtD and MfAgo‐gDtR complexes. Colors represent the magnitude of structural displacement between the two states, ranging from blue (0 Å) to red (2 Å). (c) Structural superposition of the DNA‐RNA duplex in the cleaved states of TtAgo‐gDtR, KmAgo‐gDtR and the monomeric MfAgo‐gDtR complex. TtAgo‐gDtR and KmAgo‐gDtR are shown in gray ribbons. (d,e) Detailed interactions between the PIWI loops and the nucleic acid duplex in MfAgo‐gDtD (d) and MfAgo‐gDtR (e). (f) Cleavage activity analysis of wild‐type MfAgo and its mutants toward DNA and RNA substrates.

Given that dimerization is required for catalytic tetrad occupation during MfAgo‐mediated RNA cleavage, we investigated whether this oligomeric assembly is equally essential for DNA cleavage. Strikingly, both group mutants 1 and 2 retain DNA cleavage capability independent of dimerization (Figure S8c). To pinpoint the key residues driving the phenotype within group mutant 2, we further introduced single‐point mutation F30A, which inserted into the corresponding pocket responsible for the binding of the original 3’‐end guide DNA (Figure S7e, f). Notably, F30A largely disrupted dimerization and completely abolished RNA cleavage, yet its DNA cleavage activity retained. Moreover, F30W, which partially impair dimerization, only showed a correspondingly moderate reduction in RNA cleavage (Figure S8e,g). Collectively, these results further support that dimerization is specifically required for RNA cleavage but dispensable for DNA cleavage, revealing a divergence between MfAgo‐mediated DNA and RNA cleavage. Besides, as the cryo‐EM dataset of the MfAgo‐gDtD complex exclusively comprised dimeric particles, this dimeric state may represent a highly stable conformation in which product release hindrance constitutes the rate‐limiting step. Multiple‐turnover kinetic analyses revealed that wild‐type MfAgo exhibits a low overall turnover rate for DNA cleavage with the products accumulating slowly (Figure 5a). In contrast, RNA cleavage appears complete catalytic cycle with markedly higher efficiency after the initial lag phase (Figure 5a). Furthermore, these monomeric mutants retain multiple‐turnover rates compared to wild‐type for DNA cleavage, indicating that the low multiple‐turnover rate arises from the intrinsic differences in nucleic acid hybrid stability rather than dimerization state of MfAgo (Figure S8f). Therefore, these findings suggest that the mix of the monomeric and dimeric states of in the RNA‐targeting dataset may reflect the rapid turnover of RNA cleavage, allowing multiple oligomeric states to be captured during cryo‐EM analysis. In contrast, the stable dimeric conformation observed in the MfAgo‐gDtD dataset may represent a more stable cleavage complex associated with cleaved DNA product.

To comprehensively elucidate the structural basis of these divergent cleavage mechanisms, we compared the cleaved ternary structures of MfAgo‐gDtD and MfAgo‐gDtR and identified two distinct rearrangements within the nucleic acid duplex and the PIWI‐loop region (Figure 5b). In MfAgo‐gDtR, the 5’‐cleaved RNA fragment undergoes a pronounced outward flip unlike the canonical guide‐target duplex observed in TtAgo (PDB:3HK2) [31] and KmAgo (PDB:8XJW) [32], facilitating rapid dissociation of the RNA product (Figure 5c). In contrast, the cleaved DNA product remains rigidly anchored within the dimeric MfAgo‐gDtD complex (Figure S9a). Besides, the nucleic acid duplex of MfAgo‐gDtD also highly resemble the pre‐cleavage intermediate 2 captured in MfAgo‐gDtR, suggesting that the DNA product is hindered to undergo the conformational transition required for product dissociation (Figure S9b). This structural divergence is governed by the distinct rearrangement of the PIWI‐loops, which simultaneously modulate both substrate binding and the dimerization interface. In MfAgo‐gDtD, PIWI‐loop1 (G495‐V508) establishes an extensive polar interaction network with the DNA duplex through residues K504 and R507, whereas the corresponding region in MfAgo‐gDtR forms only a single interaction between F503 and target nucleotide 9 (Figure 5d,e and Figure S9c). Additionally, residue E534 in PIWI‐loop2 (P526‐P537) of MfAgo‐gDtD interacts favorably with R562, while the corresponding position in MfAgo‐gDtR presents an opposing electrostatic potential (Figure 5d,e). These protein‐DNA contacts, together with the stabilization of the PIWI‐loop conformation, provides a structural rationalization for the kinetic barrier across the catalytic cycle under the intrinsically low multiple‐turnover rate observed for DNA cleavage. Beyond substrate contacts, the PIWI‐loop region also engaged with remodeling the dimerization interface in a substrate‐dependent manner. MfAgo‐gDtD exhibits a stable *π*–*π* interaction containing F503‐P532’ at the dimerization interface, while in MfAgo‐gDtR, PIWI‐loop1 instead contributes two polar interactions including N506‐S531’ and K504‐E534’ (Figure S9d). Structural analysis reveals that MfAgo‐gDtD exhibits a substantially higher hydrophobic burial at the dimerization interface (∼37%) compared with MfAgo‐gDtR (∼23%), despite their identical protein sequences (Figure S9e). To functionally validate these structural findings, we introduce alanine substitutions at key PIWI‐loop1 residues. F503A and R507A severely impaired RNA cleavage while only slightly reducing DNA cleavage activity, underscoring the strict structural requirements for RNA processing (Figure 5f). Interestingly, the R535A mutant both enhanced DNA and RNA cleavage activity, potentially by alleviating steric hinderance and facilitating stabilization of the catalytic tetrad (Figure 5f). Collectively, these structural and mutational analyses demonstrate that MfAgo possesses dimerization‐independent catalytic robustness for DNA cleavage with low multiple‐turnover rate arising from intrinsic protein‐substrate interactions and PIWI‐loop stabilization that impedes conformational rearrangement. Conversely, dimerization‐induced remodeling of PIWI‐loop region not only establishes the requirement for efficient RNA cleavage capability but also rapid product dissociation.

### 2.6 An Auxiliary Nucleic Acid‐Binding Pocket Mediates the Formation of an Inter‐Lock Conformation and the Target Binding Stabilization

**FIGURE 6:**
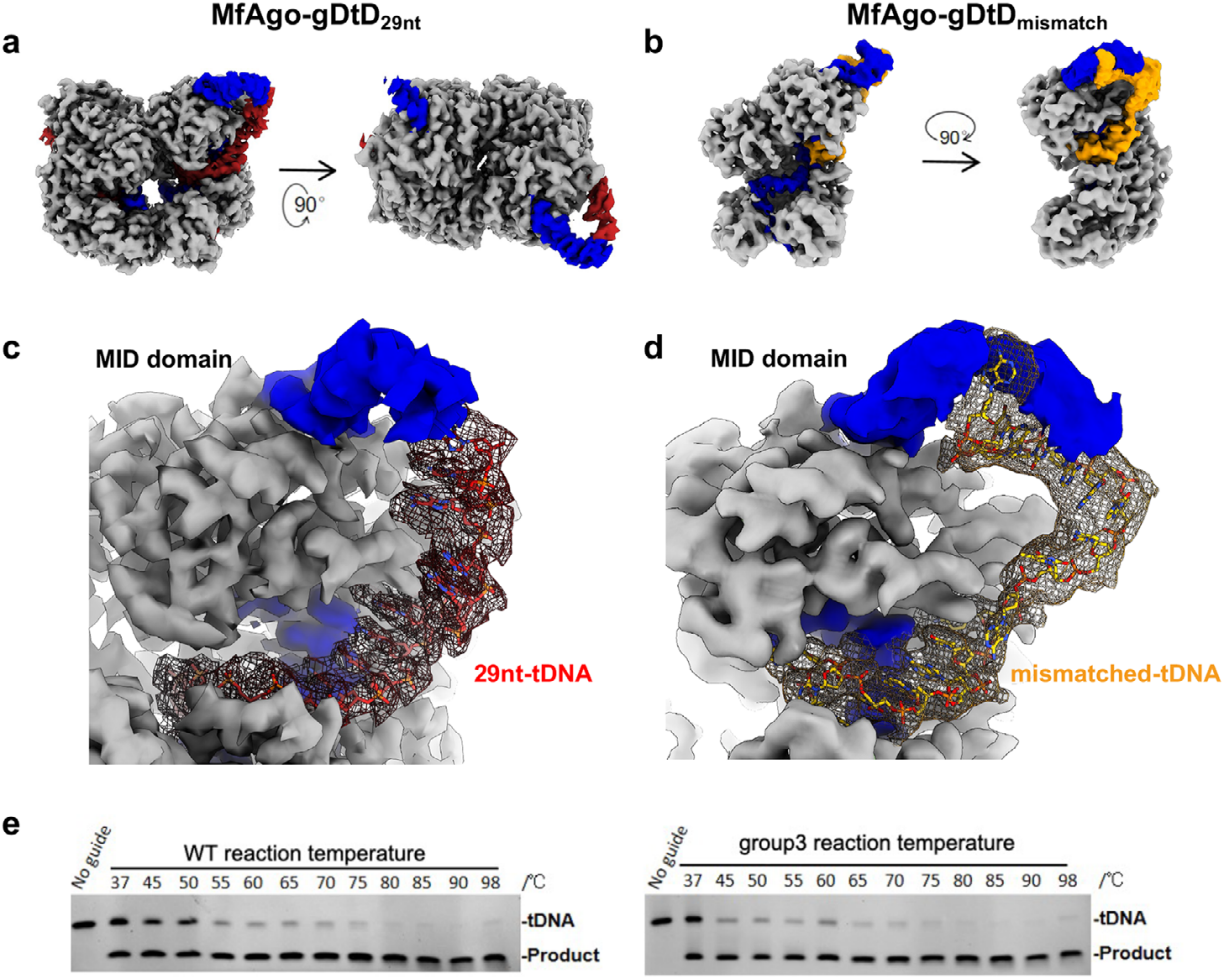
Characterization of an additional nucleic acid‐binding pocket (a) Cryo‐EM density map of the dimeric MfAgo‐gDtD_29nt_. Densities corresponding to the target strand and the additional nucleic acid are shown in red and blue, respectively. (b) Cryo‐EM density map of the monomeric ternary complex of MfAgo‐gDtD_mismatch_. The random nucleic acid (golden yellow) is partially paired with the guide DNA. Extra nucleic acid density located near the MID domain and the guide DNA is colored in blue. (c,d) Volume filtered density maps and corresponding structures of the extended 29 nt target DNA (c) and mismatched target DNA (d), shown as mesh and colored in red and golden yellow, respectively. (e) Cleavage assays of wild‐type MfAgo and mutants of the additional nucleic acid‐binding cavity against DNA substrates.

An unassigned density adjacent to the MID domain was observed in both binary and ternary complexes, which we hypothesized to correspond to a random nucleic acid fragment stabilized by stacking interactions involving K368, Y372 and F392 (Figure 3f). To test whether this auxiliary pocket participates in target pre‐binding, we determined the cryo‐EM structure of MfAgo‐gDtD complex with an extended 29‐nt target strand (MfAgo‐gDtD_29nt_, 2.6 Å) (Figure S10). Although limited local resolution at the distal region precluded unambiguous modelling, a continuous density extending from the guide‐target duplex into the auxiliary pocket was clearly distinguishable. This observation strongly suggests that the pocket anchors and stabilizes the target strand during cleavage (Figure 6a,c). Furthermore, to mimic the complex in vivo environment where MfAgo frequently encounters an excess of non‐cognate ssDNA, we captured cryo‐EM maps of MfAgo incubated with partially complementary targets (MfAgo‐gDtD_mismatch_, 3.1 Å) in monomeric conformations (Figure 6b and Figure S11a–d). We found that when an off‐target DNA substrate shares limited complementary solely within the seed region, MfAgo adopts a locked conformation that arrests further duplex propagation and precludes complete target association (Figure 6d). This trapped state in MfAgo may underpin the requirement for elevated reaction temperatures to dissociate the off‐target dsDNA duplex. Therefore, these structural observations indicate that the auxiliary pocket provides additional stabilization to the target strand, which can also stabilize non‐productive intermediates during off‐target recognition.

To validate the specific role of this auxiliary pocket, we engineered group mutations (group mutant 3) within the auxiliary nucleic acid cavity. Based on our electrophoretic mobility shift assays (EMSA) assays, the disruption of the auxiliary pocket diminished the target binding, indicating the auxiliary pocket contributes to stable target association (Figure S12a). Consistent with this observation, in vitro cleavage assays demonstrated that disrupting this pocket significantly narrowed the optimal temperature range for RNA cleavage with a decrease activity at 75°C (Figure S12b). As MfAgo‐mediated RNA cleavage is highly sensitive, the disruption of auxiliary pocket may diminish the conformational stability at elevated temperatures, thereby reducing the cleavage efficiency. Interestingly, DNA cleavage of the group mutant 3 is highly increased at 45°C and 50°C (Figure 6e). In contrast to RNA substrates, DNA cleavage by MfAgo is less dependent on additional target stabilization, while the DNA‐targeting complex is intrinsically stable and highly component to perform cleavage (Both monomer and dimer conformation are capable to target DNA, and the dimer conformation is more feasible to form than RNA‐targeting complex). Under these conditions, the stabilization provided by the auxiliary pocket may instead over‐stabilize the DNA‐bound complex, while the disruption of the auxiliary pocket is capable to relieve the excessive stabilized target and thereby facilitate productive DNA cleavage.

Collectively, these structural and biochemical assays suggest that the auxiliary pocket serves as an element for target recognition by providing additional target stabilization. The stabilization is favorable for structurally sensitive RNA substrates, while for the intrinsically stable DNA target, it seems less beneficial and even impede its efficient cleavage. Conversely, as DNA substrates are readily recognized by MfAgo, the auxiliary pocket may stabilizes interlocked off‐target intermediates, thereby preventing cleavage among mismatched DNA.

## 3 Discussion

To date, prokaryotic Argonaute system presents high sensitivity and sequence‐specific targeting, emerging as a new generation in diagnosis platform. However, most Argonaute‐based applications depend on DNA‐guided DNA cleavage activity by hyperthermal Argonautes, resulting a general lack of practical substrate versatility and the strict dependence on elevated reaction temperature. Although engineered mutants of hyperthermophilc PfAgo exhibit additional RNA cleavage, they are limited by low catalytic efficiency and mismatch tolerance. In this study, we characterized a novel pAgo from *Methanocaldococcus fervens*, revealing an intrinsic RNA cleavage capability alongside its canonical DNA targeting activity under a wide range of reaction temperature with high accuracy. Besides, MfAgo processes notably substrate versatility, including highly structured RNAs and double‐strand plasmids. This demonstrates that MfAgo may represent a highly promising tool in nucleic acids manipulation.

Through systematically determining the optimal biochemical parameters for RNA cleavage and resolving the comprehensive series of functional states along the catalytic activity targeting DNA and RNA, we demonstrate a series of conformational rearrangement during guide and target engagement, while DNA and RNA induce divergent remodeling of the ternary complex conformation. Two pre‐cleavage and two product‐release intermediates along RNA cleavage pathway were identified and structural analysis of these intermediates delineates stepwise progress from initial target capture to cleavage and subsequent product dissociation. Notably, the PIWI‐loop region, especially the catalytic glutamate finger E534, fail to achieve a stable active conformation in the absence of the dimerization constraints. E534 remains highly flexible or orients outward from the active site through the cleavage pathway, however, its alanine mutant (E534A) completely abolishes RNA cleavage activity, DNA cleavage is retained, albeit with markedly reduced efficiency, highlighting the high structural tolerance inherent to DNA cleavage (Figure S9f). Given that the MfAgo‐gDtR complex captures a pre‐release state featuring an outward‐shifted the 5’‐cleaved product, dimerization may promote a transient insertion of E534 into the active site and perform an outward movement corresponding with the product release. The dimerization requirement for cleavage has now been shown among characterized pAgo, such as PfAgo, CpAgo and VbAgo, while self‐assembly directly gates the nuclease active site in a substrate‐dependent manner [7, 8, 9]. However, whereas dimerization is strictly required for RNA cleavage, MfAgo mutant‐mediated DNA cleavage proceeds efficiently in the monomeric form, presenting divergent dependence on dimerization. Structural comparison between the DNA‐ and RNA‐bound states reveals that the PIWI‐loop region adopts substrate‐dependent conformation in a distinct manner. The polar contact network between PIWI‐loop region and the DNA duplex anchors the loop into a rigid conformation that may impose a kinetic barrier across the catalytic cycle, while the flexible remodeling of the PIWI‐loop during RNA cleavage facilitates rapid conformation rearrangement and efficient product dissociation after the initial lag phase. As pAgos represent a typical defense system, they are likely maintained at a relatively low baseline abundance under physiological conditions. Upon invasion, foreign nucleic acids may recruit guide‐loaded MfAgo to execute its defensive function. It is therefore conceivable that, as DNA cleavage is independent of dimerization, the MfAgo‐gDNA complex can cleave DNA substrates immediately after target recognition. In contrast, the kinetic lag phase observed during RNA cleavage likely reflects the time required for target‐induced stable dimerization, which consequently triggers the necessary conformation rearrangement for cleavage activity. Taken together, these findings establish that the same structural element of MfAgo plays a substrate‐dependent role during cleavage.

Furthermore, we identified an auxiliary cavity within the MID domain of MfAgo accommodated with non‐specific single‐strand nucleic acid, similar to that observed in PfAgo. Our structural and biochemical analyses suggest that this pocket participates in the substrate recognition by providing additional target stabilization with a substrate‐dependent consequence. This stabilization is essential for the formation of functional RNA‐targeting complexes, while for the intrinsically stable DNA‐targeting complex it may seem excessive, restricting the cleavage efficiency. Additionally, we discovered that it may also render the cleavage capability susceptible to inhibit by the random nucleic acids. These random nucleic acids, which partially pair with the seed region of the guide, can trap the MfAgo binary complex in a locked conformation, thereby physically preventing the central cleft from binding a full complementary target. We propose that the MID‐mediated self‐locking mechanism constitutes an intrinsic quality‐control checkpoint, showing temperature‐dependent fidelity and providing a structural explanation for the high‐temperature dependency characteristic of Argonaute system. It is supposed that the high‐temperature requirement of thermophilic pAgos may characterized by its need for a loosely packed conformation at phycological temperature and efforts toward cold adaptation have explored diverse strategies among buffer optimization, protein‐specific point mutagenesis and deep‐learning‐guided sequence design [24, 33, 34]. Mutations within the MID cavity facilitate DNA cleavage at room temperature by disrupting the self‐locking, offering a rational engineering strategy for cold‐adapted Argonaute applications.

Overall, our findings provide a comprehensive view of the cleavage capabilities and mechanisms of MfAgo across diverse substrates, presenting it as a promising programmable nuclease for gene manipulation and molecular diagnostics.

## 4 Methods

### 4.1 Phylogenetic Tree Analysis

Protein homologs of MfAgo were identified by BLASTp searches against the NCBI non‐redundant (nr) protein database. Representative pAgo protein sequences were retrieved from the NCBI database and aligned using ClustalW implemented in MEGA12. A phylogenetic tree was constructed using MEGA12.

### 4.2 Protein Expression and Purification

The sequence of Argonaute from *Methanocaldococcus fervens* (WP015791216.1) was codon‐optimized for *Escherichia coli* and cloned into the pET28a vector with N‐terminal 8*His tag. Mutants were constructed using the polymerase chain reaction with primers containing the single mutations, followed with homologous recombination mediated by T5 exonuclease. MfAgo and mutants were expressed in *E. coli* BL21(DE3) and cultivated in LB medium containing 50 µg/mL kanamycin (Kana) at 37°C until the OD_600_ reaching a value between 0.6 to 0.8, followed with the induction using the isopropyl β‐D‐1‐thiogalactopyranoside (IPTG) for 17 h in 18°C. The cells were isolated and resuspended in the purification buffer of 50 mM Tris, pH 7.5, 500 mM NaCl, 5% (w/v) Glycerol, 5 mM MgCl_2_, 1 mM PMSF. The resuspension was treated with high‐pressure homogenizer for bacterial cell disruption, and the supernatant was collected after centrifuged at 17 000 rpm for further purification. The Ni‐NTA column was pre‐washed with the purification buffer and incubated with the lysate supernatant for 50 min in 4°C. The column was eluted sequentially with buffers containing a gradient concentration of imidazole, such as 20, 50, and 400 mM. The eluted proteins were desalted using G‐25 desalting colume (Cytiva) equilibrated in desalting buffer, containing 20 mM Hepes, pH 7.5, 300 mM NaCl, 1 mM DTT. To remove the endogenous nucleic acids, the proteins were loaded onto the Heparin Focurose 6FF column (Cytiva), which was pre‐washed with desalting buffer and eluted with a linear NaCl gradient from 0.1 to 2 M. The eluate was concentrated using 50 kDa molecular weight cut‐off (MWCO) centrifugal filter units and further purified by size‐exclusion chromatography (SEC) using the storage buffer (20 mM Hepes, pH 7.5, 150 mM NaCl, 1 mM DTT). The highly purified proteins were analyzed by SDS‐PAGE and flash‐frozen in liquid nitrogen as well as stored at −80°C.

### 4.3 Single Strand Nucleic Acid Cleavage Assays

The single strand DNA and RNA cleavage assays were performed at a molar ration of 10:2:1 (MfAgo:guide:target) and final concentration of 2 µM MfAgo and 0.4 µM guide were pre‐incubated at 37°C for 10 min in reaction buffer of 10 mM Hepes, pH 7.5, 250 mM NaCl, 5% Glycerol, 5 mM divalent ions. Subsequently, the cleavage reaction was initiated by adding 0.2 µM target nucleic acid. For the DNA target, the mixture was incubated at 85°C for 10 min, whereas the reaction targeting RNA was conducted at 60°C for 20 min. All reactions were terminated with the equal volumes of 2×RNA loading buffer (95% (w/v) Formamide, 18 mM EDTA, 0.025% (w/v) SDS, 0.025% (w/v) Bromophenol blue). After a heating at 95°C for 5 min, the samples were detected using 20% Urea‐PAGE and visualized by Gel Doc XR+ (Bio‐Rad, California, USA), analyzed by ImageJ and GraphPad Prism software.

### 4.4 Multi‐Turnover Kinetic Assays

For the multi‐turnover assays, MfAgo and its mutants were pre‐incubated with guide DNA at a molar ratio of 1:2 (protein:guide) at 37°C for 10 min to generate the binary complex assembly. DNA cleavage reactions were then performed at 85°C, while RNA cleavage conducted at 60°C, at a final molar concentrations of 300, 600, 1500 nM for protein, guide, and target, respectively. Reaction samples were terminated at 0, 3, 6, 9, 12, 15, 30, 45, 60, 75, 90, 105, and 120 min after reaction initiation. Reaction products were resolved on 20% Urea‐PAGE and visualized by Gel Doc XR+ (Bio‐Rad, California, USA). Data quantified by ImageJ were subjected to GraphPad Prism software to determine the cleavage kinetics. DNA cleavage data were fitted to a burst kinetics equation “Y = B ^*^ (1 – exp(‐kburst ^*^ X)) + V ^*^ X”. RNA cleavage data were fitted to a one‐phase exponential model with a lag time (X_0_) “Y = SteadyState^*^(1‐exp(‐K^*^(X‐X0)))+Baseline for X ≥ X0)”.

### 4.5 High‐Structured Nucleic Acid Cleavage Assays

For plasmid cleavage, 2 µM MfAgo were incubated with guides targeting the plasmid at opposite site (F and R guides) containing various GC contents (29%, 39%, 45%, 53%, and 65%) at 37°C for 10 min in the plasmid reaction buffer (10 mM Hepes, pH 7.5, 250 mM NaCl, 5% (w/v) Glycerol and 0.2 mM MnCl_2_). Plasmids (1600 ng pUC19) were loaded for cleavage analyses at 85°C for 20 min and the reaction were terminated with the loading of the 6×DNA loading buffer (30 mM EDTA, 36% (w/v) Glycerol, 0.05% (w/v) Xylene Cyanine Blue FF and 0.05% (w/v) Bromophenol blue). Samples were subjected to 0.7% agarose gel and visualized by Gel Doc XR+ (Bio‐Rad, California, USA) for further analysis.

The HIV‐1 ΔDIS 5’UTR [29, 35] was in vitro transcribed using TranscriptAid T7 High Yield Transcription kit (Thermo Scientific), followed with DNase I treatment to eliminate template DNA and ethanol precipitation. For the cleavage assays, 2 µM MfAgo was pre‐incubated with DNA guide (400 µM) in corresponding reaction buffer (10 mM Hepes, pH 7.5, 250 mM NaCl, 5% (w/v) Glycerol and 0.2 mM MgCl_2_) at 37°C for 10 min for guide loading. HIV‐1 ΔDIS 5’UTR was introduced to a final concentration of 200 µM and the cleavage was carried out at 60°C for 30 min. The samples were quenched by adding an equal volume of 2×RNA loading buffer and denatured at 95°C for 5 min. Cleavage products were resolved by 10% Urea‐PAGE and analyzed consistent with the single‐strand cleavage assay.

### 4.6 Native Polyacrylamide Gel Electrophoresis (Native‐PAGE)

To evaluate the oligomeric state of MfAgo‐gD and MfAgo‐gDtD, purified wild‐type MfAgo and its mutants (1 mg/mL) were incubated with guide DNA at a 1:1 molar ratio at 37°C for 10 min to assemble the binary complex. To form the ternary complex, the pre‐assembled binary complex was further incubated with the target DNA (protein‐guide:target = 2:1) at 85°C for 20 min. The samples were then resolved on a 4% native PAGE gel (Beyotime Biotechnology), and the gel was stained with Coomassie Brilliant Blue.

### 4.7 Assembly of MfAgo‐Related Complexes

All the guide and targets were dissolved in DEPC‐treated water. For the assembly of the MfAgo‐gD, MfAgo were incubated with 18 nt gDNA at 37°C for 10 min in the DNA reaction buffer (10 mM Hepes, pH 7.5, 250 mM NaCl, 5% (w/v) Glycerol and 5 mM MnCl_2_) and RNA reaction buffer (10 mM Hepes, pH 7.5, 250 mM NaCl, 5% (w/v) Glycerol and 5 mM MgCl_2_). The ternary complex of MfAgo‐gDtD and MfAgo‐gDtR were obtained in a 10 min reaction with the 19 nt DNA and RNA target loaded into the reaction mixture of MfAgo‐gD at 85°C and 60°C, respectively, while the MfAgo:gDNA:tDNA/tRNA at a molar ratio of 2:2:1. The ternary complex of MfAgo loaded with mismatched target DNA were prepared through the same procedure.

### 4.8 Cryo‐EM Sample Preparation

Aliquot of 4 µL sample of MfAgo‐related complexes was loaded onto the glow‐discharged 400‐mesh R1.2/1.3 Au grids (Quantifoil) at 4°C under 100% humidity with the wait time of 5 s and the blot time of 4 s. Using the Vitrobot Mark IV (Thermo Fisher Scientific), the grids were plunge‐freezed into the liquid methane and transferred into the liquid nitrogen for further data collection and storage.

### 4.9 Cryo‐EM Data Collection and Data Processing

All samples were examined by Titan Krios microscope operated at 300 kV, equipped with Gatan K3 camera and Quantum energy filter. Datasets were collected at a magnification of 105 000× with a defocus range from −1.0 to −1.5 µm and a total dose of 47 e^−^ using the EPU software.

All the datasets were performed using CryoSPARC software [36]. For the processing of apo and binary complex of MfAgo, 4592 and 4507 micrographs were collected and subjected to motion correction and CTF parameters estimation [37]. Particles were picked for the initial template creation through multiple rounds of ab‐initio refinement and hetero refinement. Good classes were selected as the following template for the template picking, while 7 457 125 and 7 001 154 particles were collected for the further refinement, respectively. After four rounds of ab‐initio refinement and hetero refinement, classes with good features of Argonaute were chosen and refined for the final 3D classification and Nu refinement. The final densities of MfAgo‐apo and MfAgo‐gD were identified at a resolution of 3.16 and 3.30 Å, respectively.

For the further processing of the ternary complex of MfAgo‐gDtD, 3,899 micrographs were collected and 7 029 283 particles were selected for ab‐initio refinement and hetero refinement using blob picker, followed with 2D classification. Good classes were gathered for further refinement, and the resolution of the final map were estimated as 2.74 Å with the 0.143 criterion of the Fourier shell correlation curve by CryoSPARC. For the datasets of MfAgo loaded with guide DNA and target RNA, 4233 movies were obtained and followed with motion correction and CTF estimation. 3,629,973 particles were picked for multiple ab‐initio refinement, hetero refinement and 2D classification. A mixture of monomer and dimer conformation can be distinguished during the classification and 195 053 particles with clear feature of dimerized conformation were further processed with Nu‐refinement at a final resolution of 2.97 Å. As for the monomer conformation in the dataset processing, the density map of MfAgo‐gD were selected as the initial template for the monomer particle picking and 6 432 670 particles were subjected to two rounds of ab‐initio refinement and hetero refinement. According to several rounds of 3D classification, monomer density maps representing different states during MfAgo cleavage can be identified and the final maps were estimated at a resolution range from 3.16 to 3.44 Å for further analyses. Dataset of MfAgo loaded with extended target and mismatched target were processed with multi‐rounds of ab‐initio refinement and hetero refinement and a final resolution of 2.6 and 3.1 Å was obtained after the 3D classification and Nu‐refinement, respectively.

### 4.10 Model Building and Refinement

The initial model of MfAgo in the apo state was predicted using AlphaFold3 and rigid‐body docked into the density map using UCSF Chimera [38, 39]. The pre‐fitted model was further manually adjusted using Coot and subjected to real‐space refinement in Phenix [40, 41]. The guide and target nucleic acids were build de novo in Coot based on their sequences and manually fitted into the density. Iterative rounds refinement in Coot and Phenix facilitates the acquisition of the final coordinate files. The structural parameters were validated via MolProbity in Phenix, and all structural presentations were generated using UCSF Chimera and ChimeraX [42].

### 4.11 Electrophoretic Mobility Shift Assays (EMSA)

EMSA were performed to access the binding of wild‐type MfAgo and its mutant to DNA and RNA substrate. Wild‐type MfAgo and its mutant were incubated with a fixed concentration of DNA guide (400 nM) at molar ratios of protein:guide of 1:80, 1:20, 1:4, and 1:2. The mixture were incubated in cleavage‐deficient buffer (10 mM Hepes, pH 7.5, 250 mM NaCl, 5% Glycerol, 5 mM CaCl_2_) at 37°C for 10 min. The mixtures were then incubated with the DNA and RNA substrate adding at a fixed concentration at 400 nM at 75°C for 10 min. Samples mixed with EMSA loading buffer (25 mM Tris, pH 7.5, 4% (w/v) Glycerol, 0.025% (w/v) Bromophenol Blue) were resolved on 8% Native‐PAGE and visualized by Gel Doc XR+ (Bio‐Rad, California, USA) for further analysis.

### 4.12 MfAgo‐Mediated Molecular Cloning

Two parent plasmids (pET28a‐sfGFP‐target gene and pET23a‐random gene) were constructed, providing the target gene for detection and the vector, respectively. Two pairs of 35 bp homologous sequences were designed around both the target gene fragment and the vector to generate blunt ends with identical 15 bp overlapping regions for following cleavage for MfAgo.

To isolate the target gene fused with sfGFP, two pairs of 18 nt forward and reverse guides (F1/R1 and F2/R2) were designed within the homologous regions. Each pair of oligonucleotides were responsible for the cleavage of one site in the plasmid. For cleavage reaction, 2 µM MfAgo was pre‐incubated with 1 µM of each oligonucleotide (including F1, R1, F2 and R2) at 55°C for 10 min, which were pooled for following cleavage. An aliquot of 2 µL was loaded into 8 µL plasmid 1 (pET28a‐sfGFP‐target gene), followed by cleavage at 85°C for 10 min. For the cleavage of the vector, F3/R3 and F4/R4 were designed and subjected with the same procedure above. The cleavage products were analyzed via 0.7% agarose gel electrophoresis to verify the cleavage accuracy and gel‐purified for the following plasmid construction. Fragments were treated with T5 exonuclease and transformed into BL21(DE3). The number of green fluorescent colonies were quantified and further validated by sequencing.

## Author Contributions

S.W. and J.W. conceived and supervised the study. F.H., T.C. and M.F. performed molecular cloning, protein expression, purification and cleavage assays. K.M., Y.B., and S.X. assisted with the protein purification of the mutants and cleavage assays. T.C. and X.T. assisted with the cleavage assays and data analyzation. F.H., M.F. and Q.D. performed cryo‐EM sample preparation and data collection. J.W. and F.H. determined the cryo‐EM structures and built the atomic models. S.W., J.W. and F.H. analyzed the data and wrote the manuscript. All authors participated in discussion and revised the manuscript.

## Conflicts of Interest

The authors declare no conflicts of interest.

## Supporting information




**Supporting File 1**: advs77620‐sup‐0001‐SuppMat.docx.

**Supporting File 2**: advs77620‐sup‐0002‐SuppMat.docx.

**Supporting File 3**: advs77620‐sup‐0003‐SuppMat.xlsx.

**Supporting File 4**: advs77620‐sup‐0004‐SuppMat.xlsx.

## Data Availability

The data that support this study are available in this article, and its supplementary information. The cryo‐EM maps and atomic coordinates generated in this study have been deposited into the Electron Microscopy Data Bank (EMDB) and Protein Data Bank (PDB), respectively. The PDB accession codes are as follows: 27AT (MfAgo‐apo); 27SL (MfAgo‐gD); 27AV (MfAgo‐gDtD); 27AW (MfAgo‐gDtR); 27SM (MfAgo‐gDtR_intermediate1_); 27AY (MfAgo‐gDtR_intermediate2_); 27AZ (MfAgo‐gDtR_releasing1_); 27SN(MfAgo‐gDtR_releasing2_). The EMDB codes are as follows: EMD‐81017 (MfAgo‐apo); EMD‐81387 (MfAgo‐gD); EMD‐81019 (MfAgo‐gDtD); EMD‐81022 (MfAgo‐gDtR); EMD‐81389(MfAgo‐gDtR_intermediate1_); EMD‐81024 (MfAgo‐gDtR_intermediate2_); EMD‐81025 (MfAgo‐gDtR_releasing1_); EMD‐81390 (MfAgo‐gDtR_releasing2_), EMD‐81020 (MfAgo‐gDtD_mismatch_), and EMD‐81021 (MfAgo‐gDtD_29nt_). In addition, the accession codes of the previous published structural models for comparison used in this study are: 8JPX (PfAgo‐gDtD); 9JH9 (CpAgo‐gDtD); 3HK2 (TtAgo‐gDtR); and 8XJW (KmAgo‐gDtR).
