# Supplementary material for: Distinct Cleavage Trajectories Mediated by DNA and RNA Substrate in Argonaute From Methanocaldococcus Fervens
Source: Adv Sci (Weinh). 2026 Sep 8:e77620. Online ahead of print. doi: 10.1002/advs.77620 (PMC13554494; doi:10.1002/advs.77620)
Supplement: Supplementary file 1 [file ADVS-9999-e77620-s001.docx]

|  | **MfAgo-apo** | **MfAgo-gD** | **MfAgo-gDtD** | **MfAgo-gDtD_mismatch_** | **MfAgo-gDtD_29nt_** |
| --- | --- | --- | --- | --- | --- |
| **PDB** | (27AT) | (27SL) | (27AV) | - | - |
| **EMDB** | (EMD-81017) | (EMD-81387) | (EMD-81019) | (EMD-81020) | (EMD-81021) |
| **Data collection and processing** |  |  |  |  |  |
| Magnification | 105,000 | 105,000 | 105,000 | 105,000 | 105,000 |
| Voltage (kV) | 300 | 300 | 300 | 300 | 300 |
| Electron exposure (e^-^/Å^2^) | 54 | 54 | 54 | 54 | 54 |
| Defocus range (μm) | -1.0 ~ -1.5 | -1.0 ~ -1.5 | -1.0 ~ -1.5 | -1.0 ~ -1.5 | -1.0 ~ -1.5 |
| Pixel size (Å) | 0.851 | 0.851 | 0.851 | 0.851 | 0.851 |
| Symmetry imposed | C1 | C1 | C1 | C1 | C1 |
| Initial particle projections (no.) | 7,457,125 | 7,001,154 | 7,029,283 | 2,021,260 | 2,498,043 |
| Final particle projections (no.) | 240,960 | 105,285 | 293,936 | 207,634 | 288,073 |
| Map resolution (Å) | 3.16 | 3.30 | 2.74 | 3.17 | 2.59 |
| FSC threshold | 0.143 | 0.143 | 0.143 | 0.143 | 0.143 |
| Map resolution range (Å) | 2.65 ~ 34.66 | 2.93 ~ 47.30 | 2.44 ~ 38.88 | 2.72 ~ 49.43 | 2.20 ~ 25.10 |
| **Refinement** |  |  |  |  |  |
| Initial model used | AlphaFold3 | 27AT | 27AU |  |  |
| Model resolution (Å) | 3.30 | 3.42 | 2.85 |  |  |
| FSC threshold | 0.5 | 0.5 | 0.5 |  |  |
| Map sharpening B factor (Å^2^) | -138.4 | -137.2 | -96.1 |  |  |
| Model composition |  |  |  |  |  |
| Non-hydrogen atoms | 5,586 | 6,306 | 13,318 |  |  |
| Protein residues | 704 | 704 | 1,407 |  |  |
| Nucleotide | 0 | 20 | 74 |  |  |
| Ligands | 0 | 1 | 6 |  |  |
| *B*-factors (Å^2^) |  |  |  |  |  |
| Protein | 2.62/90.18/36.64 | 24.50/151.07/69.31 | 11.83/81.12/34.11 |  |  |
| Nucleiotide | - | 43.47/127.59/80.22 | 16.03/99.20/31.12 |  |  |
| Ligand | - | 56.21/56.21/56.21 | 17.97/29.29/21.82 |  |  |
| R.m.s. deviations |  |  |  |  |  |
| Bond lengths (Å) | 0.003 | 0.003 | 0.003 |  |  |
| Bond angles (^o^) | 0.539 | 0.530 | 0.472 |  |  |
| Validation |  |  |  |  |  |
| MolProbity score | 1.66 | 1.57 | 1.24 |  |  |
| Clashscore | 6.01 | 5.13 | 3.15 |  |  |
| Rotamer outliers (%) | 0.00 | 0.00 | 0.00 |  |  |
| Ramachandran plot |  |  |  |  |  |
| Favored (%) | 95.30 | 95.71 | 97.29 |  |  |
| Allowed (%) | 4.56 | 4.00 | 2.64 |  |  |
| Disallowed (%) | 0.14 | 0.29 | 0.07 |  |  |
|  | **MfAgo-gDtR**  **(dimer conformation)** | **Intermediate1** | **Intermediate2** | **Releasing1** | **Releasing2** |
| **PDB** | (27AW) | (27SM) | (27AY) | (27AZ) | (27SN) |
| **EMDB** | (EMD-81022) | (EMD-81389) | (EMD-81024) | (EMD-81025) | (EMD-81390) |
| **Data collection and processing** |  | | | | |
| Magnification | 105,000 | | | | |
| Voltage (kV) | 300 | | | | |
| Electron exposure (e^-^/Å^2^) | 54 | | | | |
| Defocus range (μm) | -1.0 ~ -1.5 | | | | |
| Pixel size (Å) | 0.851 | | | | |
| Symmetry imposed | C1 | | | | |
| Initial particle projections (no.) | 3,629,973 | 6,432,670 | | | |
| Final particle projections (no.) | 195,053 | 203,262 | 51,579 | 49,735 | 201,748 |
| Map resolution (Å) | 2.97 | 3.21 | 3.44 | 3.30 | 3.16 |
| FSC threshold | 0.143 | 0.143 | 0.143 | 0.143 | 0.143 |
| Map resolution range (Å) | 2.58 ~ 45.22 | 2.89 ~ 45.40 | 3.09 ~ 56.27 | 1.82 ~ 50.94 | 2.71 ~ 41.148 |
| **Refinement** |  |  |  |  |  |
| Initial model used | 27AV | 27AU | 27AW | 27AW | 27AW |
| Model resolution (Å) | 3.09 | 3.46 | 3.67 | 3.43 | 3.26 |
| FSC threshold | 0.5 | 0.5 | 0.5 | 0.5 | 0.5 |
| Map sharpening B factor (Å^2^) | -98.2 | -147.2 | -119.2 | -106.5 | -130.1 |
| Model composition |  |  |  |  |  |
| Non-hydrogen atoms | 13,338 | 6,377 | 6,537 | 6,467 | 6,455 |
| Protein residues | 1,407 | 704 | 697 | 693 | 700 |
| Nucleotide | 74 | 23 | 33 | 31 | 29 |
| Ligand | 4 | 1 | 3 | 1 | 1 |
| *B*-factors (Å^2^) |  |  |  |  |  |
| Protein | 15.55/102.23/46.04 | 22.60/120.52/66.40 | 33.70/207.40/89.61 | 27.02/155.41/70.72 | 14.29/111.37/51.29 |
| Nucleotide | 26.25/138.15/43.37 | 37.89/147.37/90.97 | 53.58/143.53/97.18 | 42.15/137.30/80.82 | 26.58/150.92/69.69 |
| Ligand | 18.53/35.17/31.28 | 44.19/44.19/44.19 | 59.00/111.03/81.89 | 38.82/38.82/38.82 | 28.45/28.45/28.45 |
| R.m.s. deviations |  |  |  |  |  |
| Bond lengths (Å) | 0.003 | 0.003 | 0.002 | 0.006 | 0.004 |
| Bond angles (^o^) | 0.496 | 0.569 | 0.546 | 0.645 | 0.595 |
| Validation |  |  |  |  |  |
| MolProbity score | 1.45 | 1.87 | 1.79 | 1.90 | 1.84 |
| Clashscore | 3.84 | 7.54 | 6.64 | 7.01 | 7.65 |
| Rotamer outliers (%) | 0.00 | 0.15 | 0.00 | 0.00 | 0.00 |
| Ramachandran plot |  |  |  |  |  |
| Favored (%) | 95.94 | 92.88 | 93.51 | 91.12 | 93.52 |
| Allowed (%) | 3.99 | 6.55 | 6.20 | 8.88 | 6.20 |
| Disallowed (%) | 0.07 | 0.57 | 0.29 | 0.00 | 0.29 |
