# Supplementary material for: Distinct Cleavage Trajectories Mediated by DNA and RNA Substrate in Argonaute From Methanocaldococcus Fervens
Source: Adv Sci (Weinh). 2026 Sep 8:e77620. Online ahead of print. doi: 10.1002/advs.77620 (PMC13554494; doi:10.1002/advs.77620)
Supplement: Supplementary file 2 [file ADVS-9999-e77620-s002.docx]

**Supplementary Figure 1 DNA and RNA cleavage capability of MfAgo** **a.** Elution profile and SDS-PAGE analysis of purified MfAgo. **b.** Sequences of the guide DNA and DNA/RNA targets used in cleavage assays. **c.** Effect of NaCl concentration on MfAgo-mediated DNA cleavage directed by different guide types. **d.** Effect of guide DNA length on MfAgo-mediated cleavage activity. **e.** Sequences of the 18-nt guide DNAs containing single guide-target mismatches. Mismatched nucleotides are highlighted in yellow. **f.** Effect of guide-target mismatches on MfAgo-mediated cleavage efficiency.

**Supplementary Figure 2 Plasmid cleavage by MfAgo loaded with guides containing diverse GC content** **a.** Cleavage analysis with 29% and 39% GC content in different reaction times. Lin and SC represent as linear and supercoiled forms. **b.** Plasmid cleavage following pre-incubation of MfAgo-guide DNA complexes at different temperatures, with all subsequent cleavage reactions performed at 85℃. **c.** Plasmid cleavage following pre-incubation of MfAgo-guided DNA complexes at 37℃, with subsequent cleavage reactions performed at different reaction temperatures. **d-e.** Effect of NaCl concentration (d) and Mn^2+^ concentration (e) on MfAgo-mediated plasmid cleavage. **f.** Effect of MfAgo:guide:plasmid molar ratio on plasmid cleavage. Lanes 1-7 correspond to molar ratios of 60:40:10, 30:20:10, 15:10:10, 7.5:5:10, 6:4:10, 5:3.4:10, and 3:2:10, respectively. Plasmids with 29% and 39% GC content are shown in the upper and lower panels, respectively. Quantification of gel bands is presented as stacked bar charts on the right.

**Supplementary Figure 3** **MfAgo-mediated rapid DNA assembly. a.** Schematic overview of the MfAgo-mediated DNA fragment assembly strategy. DNA fragments derived from plasmid 1 (pET28a-sfGFP-target gene) and plasmid 2 (pET23a-random gene) were assembled into a recombinant plasmid through a process including plasmid cleavage, T5 exonuclease treatment, and transformation into *E. coli* BL21. The corresponding guide pairs used for plasmid cleavage are labeled as F1-F4 and R1-R4. **b.** Electrophoretic verification of DNA fragments generated by MfAgo-directed plasmid cleavage. **c.** Screening of recombinant plasmid transformants by GFP fluorescence on selective medium.

**Supplementary Figure 4 Cryo-EM datasets analysis of MfAgo-apo and MgAgo-gD a.e.** Representative micrographs of MfAgo-apo (a) and MfAgo-gD (e). **b.f.** Data processing workflows for MfAgo-apo (b) and MfAgo-gD (f). **c.g.** Gold-standard FCS curves estimated using cryoSPARC for MfAgo-apo (c) and MfAgo-gD (g). **d.h.** Euler angle distributions for the final density maps of MfAgo-apo (d) and MfAgo-gD (h).

**Supplementary Figure 5 Cryo-EM datasets analysis of MfAgo-gDtD a.e.** Representative micrographs of MfAgo-gDtD (a) and MfAgo-gDtR (e). **b.** Data processing workflow for MfAgo-gDtD. **c.** Euler angle distribution for the final density map of MfAgo-gDtD. **d.** Gold-standard FCS curve estimated using cryoSPARC for MfAgo-gDtD. **f.** Representative 2D class averages of the MfAgo-gDtR particles.

**Supplementary Figure 6 Cryo-EM datasets analysis of MfAgo-gDtR a.** Data processing workflow for the MfAgo-gDtR dataset. **b.** Euler angle distribution for the final density map of the dimeric MfAgo-gDtR complex. **c.** Gold-standard FCS curve estimated using cryoSPARC for the dimeric MfAgo-gDtR complex. **d-g.** Euler angle distributions and FSC curves for intermediate 1 (**d**), intermediate 2 (**e**), releasing 2 (**f**) and releasing 1 (**g**).

**Supplementary Figure 7 Structure alignment between MfAgo and other pAgos a.** Structural comparison between the guide DNA loaded binary conformation of MjAgo and MfAgo. Colors represent the magnitude of structural displacement between the two states, ranging from blue (0 Å) to red (4 Å). **b.** Structural alignment between the guide DNA loaded binary conformation of CpAgo and MfAgo. Colors represent the magnitude of structural displacement, ranging from blue (0 Å) to red (8 Å). **c.** Sequence alignment of the L2 linker across multiple pAgos. Residues corresponding to W268 in MfAgo are highlighted in a red box. **d.** Additional nucleic acid pocket in the binary and ternary states of MfAgo, compared with the corresponding structure in PfAgo-gDtD. Cryo-EM densities of MfAgo-related complexes are shown as mesh. **e-f.** Close-up view of the interaction between the 3’-end of the guide DNA and the PAZ domain in MfAgo-gD complex **(e)**, compared with the corresponding region in the MfAgo-gDtD complex **(f)**. **g-h.** Structural alignment between the ternary complexes of MfAgo and PfAgo **(g)** and CpAgo **(h)**. The dimeric conformations are shown on the left; side views of the monomer highlighted by dashed boxes are shown on the right.

**Supplementary Figure 8 Analysis among the distinction between DNA and RNA cleavage of MfAgo a.** Dimerization interfaces formed by PIWI and MID domain and by N-terminal and PAZ domains, which were designed as group mutant 1 and 2, respectively. **b.** Native PAGE analysis of wild-type MfAgo and mutant groups 1 and 2 in binary and ternary complexes. **c.** Cleavage activity of wild-type MfAgo and mutant groups 1 and 2 against DNA and RNA substrates. **d.** Structural comparison between the predicted structures of mutant groups 1 and 2 and one subunit of the MfAgo-gDtD complex. **e.** Cleavage assays (left) and native PAGE analysis of binary and ternary complexes (right) formed by wild-type MfAgo and the F30A and F30W mutants. **f.** Multi-turnover kinetic analysis of DNA cleavage by mutant groups 1 and 2. Reactions were performed at a molar ratio of protein:gDNA:tDNA of 1:2:5 (300 nM: 600 nM: 1500 nM). **g.** Size-exclusion chromatography (SEC) profiles of MfAgo and its mutants. The monomeric and dimeric species corresponding to each elution peak are indicated above the chromatograms.

**Supplementary Figure 9 Distinction between DNA and RNA cleavage of MfAgo a.** Structural alignment of the guide-target duplex in the ternary complexes of MfAgo-gDtD, TtAgo-gDtD, and PfAgo-gDtD. **b.** Structural alignment of the guide-target duplex between MfAgo-gDtD and the intermediate 2 state of MfAgo-gDtR. **c.** Detailed protein-nucleic acid interaction networks in the MfAgo-gDtD and MfAgo-gDtR complexes. Residues are colored according to the scheme in Figure 3a. **d.** Close-up views of the distinct interfaces formed by the PIWI-loop region in the MfAgo-gDtD and MfAgo-gDtR complexes. One monomer is shown as a ribbon, whereas the other is shown as an electrostatic surface with blue and red indicating positive and negative electrostatic potentials, respectively. **e.** Comparison of hydrophobic surfaces at the dimerization interfaces of the MfAgo-gDtD and MfAgo-gDtR complexes. Hydrophobic contributions (excluding Gly) are mapped onto the molecular surface, and the percentages indicate the contribution of hydrophobic surface area to the dimerization interface, as calculated by PDBePISA. **f.** Cleavage assays of DNA and RNA substrates by MfAgo-E534A mutant. Lanes M1 and M2 indicate size markers for DNA and RNA cleavage products, respectively.

**Supplementary Figure 10 Cryo-EM dataset analysis of the tenary complex of MfAgo in complex with extended DNA target a.** Representative micrograph of the MfAgo-gDtD_29nt_ dataset. **b.** Data processing workflow for MfAgo-gDtD_29nt_. **c.** Gold-standard FCS curve estimated using cryoSPARC. **d.** Euler angle distribution for the final density map.

**Supplementary Figure 11 Cryo‑EM dataset analysis of the mismatched MfAgo ternary complex a.** Representative micrograph of the MfAgo-gDtD_mismatch_ dataset. **b.** Data processing workflow for MfAgo-gDtD_mismatch_. **c.** Gold-standard FCS curve estimated using cryoSPARC. **d.** Euler angle distribution for the final density map.

**Supplementary Figure 12 EMSA and temperature-dependent cleavage assays of wild-type MfAgo and group mutant 3. a.** EMSA results of wild-type MfAgo and group mutant 3 binding to DNA and RNA substrates at various protein:guide:target molar ratios. **b.** Cleavage assays of wild-type MfAgo and mutants of the additional nucleic acid-binding cavity against RNA substrates.

Supplementary Fig1

Supplementary Fig2

Supplementary Fig3

Supplementary Fig4

Supplementary Fig5

Supplementary Fig6

Supplementary Fig7

Supplementary Fig8

Supplementary Fig9

Supplementary Fig10

Supplementary Fig11

Supplementary Fig12
